# Diagnosis of COVID-19: Is Fever the Best Indicator of COVID-19 in Vaccinated SARS-CoV-2-Positive Adults?

**DOI:** 10.7759/cureus.68749

**Published:** 2024-09-05

**Authors:** Shinji Inaba, Shuntaro Ikeda, Yuta Fujiwara, Kyosei Sogabe, Katusji Inoue, Naoyuki Nogami, Eiichi Ishii, Osamu Yamaguchi

**Affiliations:** 1 Department of Community Medicine, Pulmonology, and Cardiology, Ehime University Graduate School of Medicine, Toon, JPN; 2 Department of Cardiology, Imabari City Medical Association General Hospital, Imabari, JPN; 3 Department of Cardiology, Pulmonology, Hypertension, and Nephrology, Ehime University Graduate School of Medicine, Toon, JPN; 4 Department of Surgery, Imabari City Medical Association General Hospital, Imabari, JPN; 5 Department of Pediatrics, Imabari City Medical Association General Hospital, Imabari, JPN

**Keywords:** covid-19, fever, public health, symptoms, vaccination

## Abstract

Objectives: Coronavirus disease 2019 (COVID-19) vaccination is highly recommended to prevent the onset and severity of severe acute respiratory syndrome coronavirus 2 (SARS-CoV-2) infection in Japan. However, the impact of COVID-19 vaccination on the manifestations or presenting symptoms of SARS-CoV-2 infection in daily clinical practice remains unclear.

Methods: This retrospective single-center study was conducted from April 2021 to July 2022 in Japan. We compared the clinical manifestations of SARS-CoV-2 infection in 636 COVID-19-positive patients who visited our outpatient fever clinic, both COVID-19-vaccinated and unvaccinated.

Results: During the study period, the COVID-19 vaccination rate at the time of infection was 77.2% (n=491/636), with a median of two doses. Most manifestations, including fever, were reduced in the vaccinated group (n=196) compared to the non-vaccinated group (n=142). The temperature at the clinic decreased significantly as the number of vaccinations increased. Fever was the most common manifestation in the non-vaccinated group (76%, n=108/142), while only 30% (n=59/196) of those who received three or more COVID-19 vaccinations experienced fever. However, sore throat and cough were observed more frequently in the vaccinated group compared to the non-vaccinated group.

Conclusion: Fever may not be a reliable indicator of SARS-CoV-2 infection in vaccinated individuals, as its frequency is significantly reduced by vaccination. However, since sore throat and cough are more frequently observed in vaccinated individuals, these symptoms could be useful for recommending COVID-19 testing even in the absence of fever, aiding in the prevention of infectious outbreaks.

## Introduction

Vaccination is recommended as the most effective means against the coronavirus disease 2019 (COVID-19) pandemic, with almost 70% of the global population having received at least one dose of a COVID-19 vaccine [1]. COVID-19 vaccination has been reported to be effective not only in preventing the onset of the disease but also in mitigating severe disease after infection with severe acute respiratory syndrome coronavirus 2 (SARS-CoV-2) [1-4].

Although COVID-19 vaccination may alleviate post-illness manifestations, the impact of vaccination on these manifestations in the vaccinated population is not fully understood. Since manifestations or symptoms are useful indicators for suspecting SARS-CoV-2 infection and for recommending testing for a definitive diagnosis, investigating the effects of vaccination on these manifestations is critically important for public health control. We, therefore, conducted this study to evaluate COVID-19 manifestations in the vaccinated general population who visited our outpatient fever clinic.

## Materials and methods

Patients and methods

This study was a retrospective single-center ‌analysis conducted at the outpatient fever clinic of Imabari City Medical Association General Hospital, Imabari, Japan, from April 2021 to July 2022. The study was approved by the Research Ethics Committee of Ehime University Graduate School of Medicine, Toon, Japan (Approval number: 2201006).

**Figure 1:**
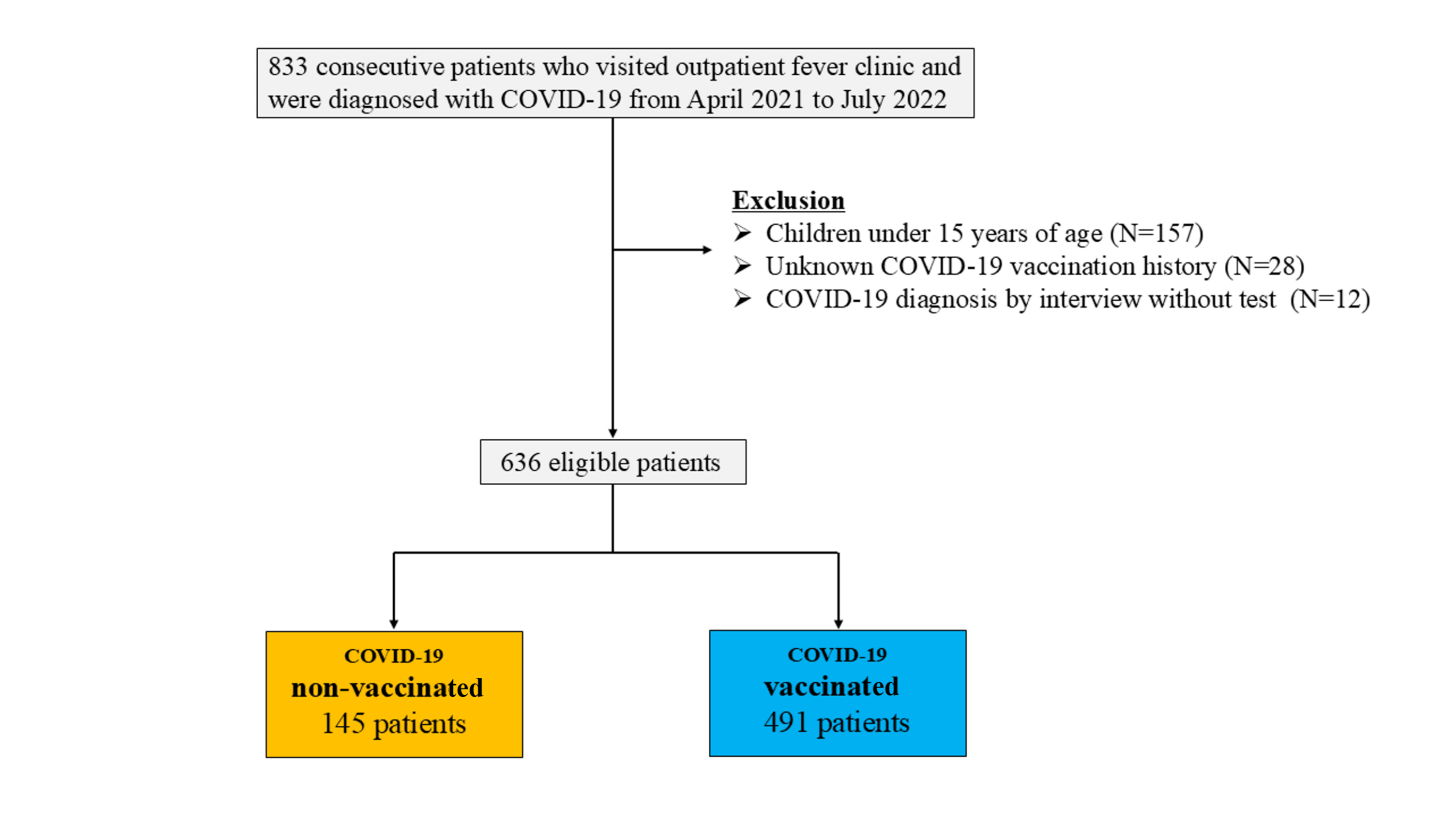
Flow chart of eligible patients. COVID-19, coronavirus disease 2019.

A total of 833 consecutive patients who visited the outpatient fever clinic and were diagnosed with COVID-19 were enrolled. From these 833 patients, we excluded 157 children under 15 years old, 28 patients with an unknown history of COVID-19 vaccination, and 12 patients diagnosed with COVID-19 based on interviews alone without testing. Ultimately, 636 patients were included in this study (Figure 1). Of these 145 were non-vaccinated and 491 were vaccinated. Of the 491 vaccinated people, nine did not know the number of vaccines taken.

Diagnosis of COVID-19 was done using polymerase chain reaction (PCR) tests and/or antigen kits from nasopharyngeal swabs. The Omicron wave was defined as the period during which COVID-19 patients were diagnosed after January 2022. The COVID-19 vaccinated group was defined as patients who had received at least one vaccination prior to their SARS-CoV-2 infection. The vaccinated subjects in the study were vaccinated by one of the following three types available in Japan: BNT162b2 (Comirnaty, Pfizer/BioNTech), mRNA-1273 (Spikevax, Moderna/Takeda), and AZD1222 (Vaxzevria, Oxford/AstraZeneca). Two of these (BNT162b2 and mRNA-1273) are mRNA vaccines, and the third (AZD1222) is a viral vector vaccine. Fever was defined in this study as a body temperature of 37.5°C or higher at the time of the hospital visit.

Statistical analyses 

Categorical variables were presented as frequencies with percentages, and continuous variables were expressed as medians and interquartile ranges. Categorical variables were compared using the chi-square test. The Mann-Whitney U test or the non-parametric Kruskal-Wallis test was applied to continuous variables. A p-value of <0.05 was considered statistically significant. Logistic regression analysis was performed to identify factors associated with COVID-19 manifestations (fever, cough, and sore throat), and we calculated odds ratios and their 95% confidence intervals. Multivariate analyses were performed after adjusting for age and gender. As confounder variables, we incorporated the number of days since symptom onset and the Omicron wave into the model because these factors may affect the frequency of symptoms. Statistical analyses were conducted using EZR (Easy R) statistical software (Saitama Medical Center, Jichi Medical University, Saitama, Japan) [5].

## Results

**Figure 2:**
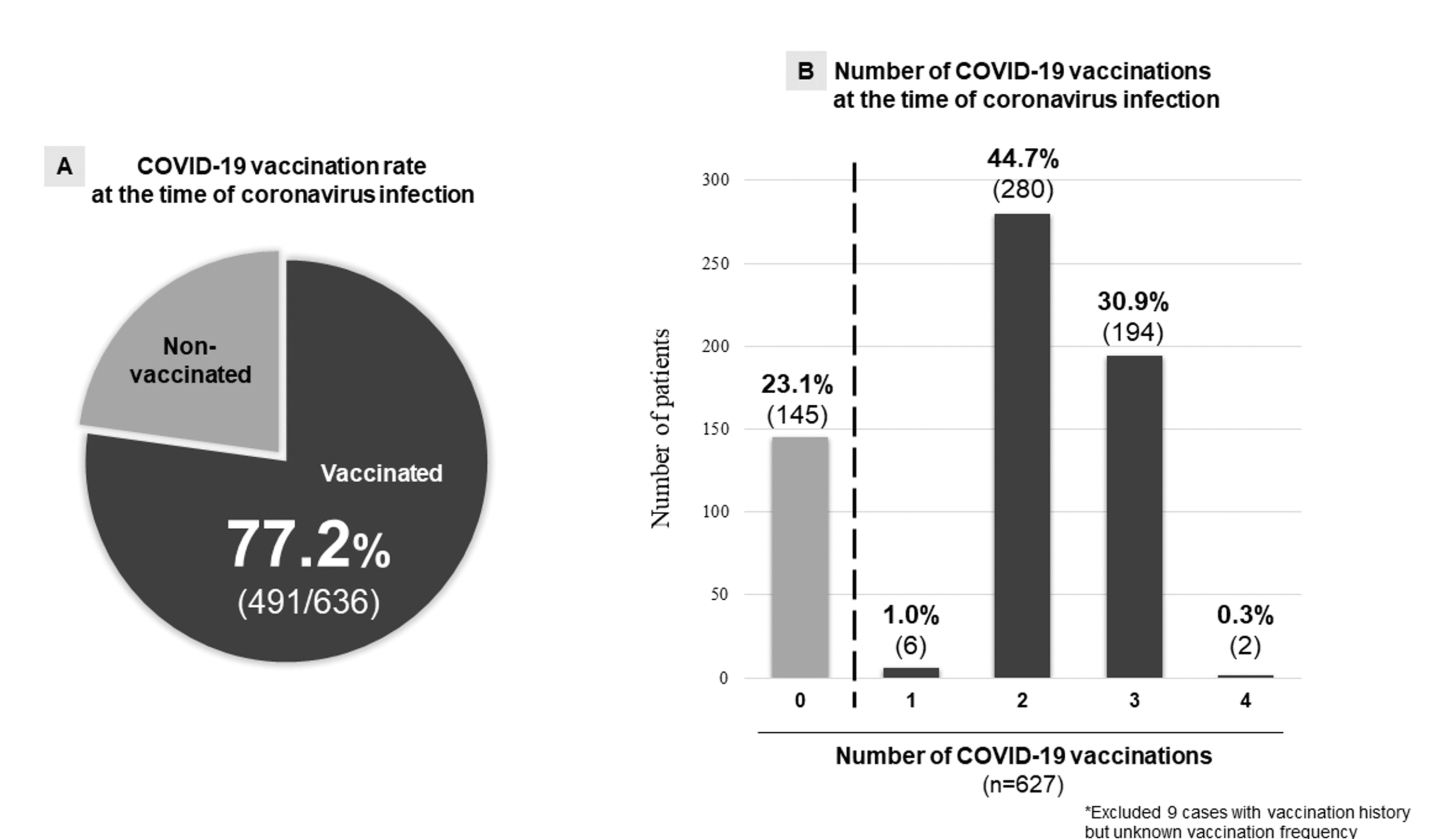
(A) COVID-19-positive subjects with and without vaccination; (B) Distribution of the vaccinated participants according to the number of vaccines taken COVID-19, coronavirus disease 2019; SARS-CoV-2, severe acute respiratory syndrome coronavirus 2

The COVID-19 vaccination rate at the time of infection was 77.2% (n=491/636), with a median of two vaccinations administered during the study period (Figure 2). Nine vaccinated patients were not sure about the number of vaccines taken. The median time from symptom onset to hospital visit was one day.

The COVID-19 vaccinated group was significantly older than the non-vaccinated group. The proportion of females was higher in the COVID-19 vaccinated group compared to the non-vaccinated group. A comparison of COVID-19 manifestations between non-vaccinated and vaccinated patients is shown in Table 1.

**Table 1:** Comparison of COVID-19 manifestations between non-vaccinated and vaccinated patients Values are median (interquartile range (IQR)) and percentage (number of observations/total number of patients) COVID-19, coronavirus disease 2019

	Vaccinated group (n=491)	Non-vaccinated group (n=145)	p-value
Age (years), median (IQR)	39 (28, 53)	33 (22, 46)	<0.001
Sex (male), % (n/N)	47.7% (234/491)	56.6% (82/145)	0.074
Time from symptom onset to hospital visit (days), median (IQR)	1.0 (0.0, 1.0)	1.0 (0.0, 2.0)	<0.05
Symptoms that were reduced in COVID-19 vaccinees			
Temperature at clinic (˚C), median (IQR)	37.4 (36.8, 38.1)	38.0 (37.5, 38.7)	<0.001
-Fever (≥37.5 ˚C at clinic), % (n/N)	231/486 (47.5%)	76.1% (108/142)	<0.001
Arthralgia, % (n/N)	8.6% (42/491)	24.8% (36/145)	<0.001
Headache, % (n/N)	37.9% (186/491)	57.2% (83/145)	<0.001
Fatigue, % (n/N)	29.5% (145/491)	38.6% (56/145)	<0.05
Nausea/vomiting, % (n/N)	6.5% (32/491)	11.7% (17/145)	0.059
Loss of appetite, % (n/N)	10.0% (49/491)	15.9% (23/145)	0.07
Gastralgia, % (n/N)	1.2% (6/491)	3.4% (5/145)	0.15
Myalgia, % (n/N)	1.8% (9/491)	4.1% (6/145)	0.20
No symptoms, % (n/N)	1.6% (8/491)	3.4% (5/145)	0.31
Shortness of breath, % (n/N)	4.5% (22/491)	6.9% (10/145)	0.34
Chills, % (n/N)	4.7% (23/491)	6.2% (9/145)	0.60
Loss of taste, % (n/N)	0.6% (3/491)	1.4% (2/145)	0.7
Loss of smell, % (n/N)	0.6% (3/491)	1.4% (2/145)	0.7
Diarrhea, % (n/N)	6.9% (34/491)	7.6% (11/145)	0.93
Constipation, % (n/N)	0.2% (1/491)	0.7% (1/145)	0.94
Cystitis symptom, % (n/N)	0% (0/491)	0% (0/415)	NA
Symptoms that were enhanced in COVID-19 vaccinees			
Sore throat, % (n/N)	79.8% (392/491)	62.1% (90/145)	<0.001
Cough, % (n/N)	67.2% (330/491)	43.4% (63/145)	<0.001
Runny nose/nasal congestion, % (n/N)	35.6% (175/491)	24.8% (36/145)	0.02
Sputum, % (n/N)	29.1% (143/491)	22.8% (33/145)	0.16
Abdominal pain, % (n/N)	3.1% (15/491)	2.8% (4/145)	1

**Figure 3:**
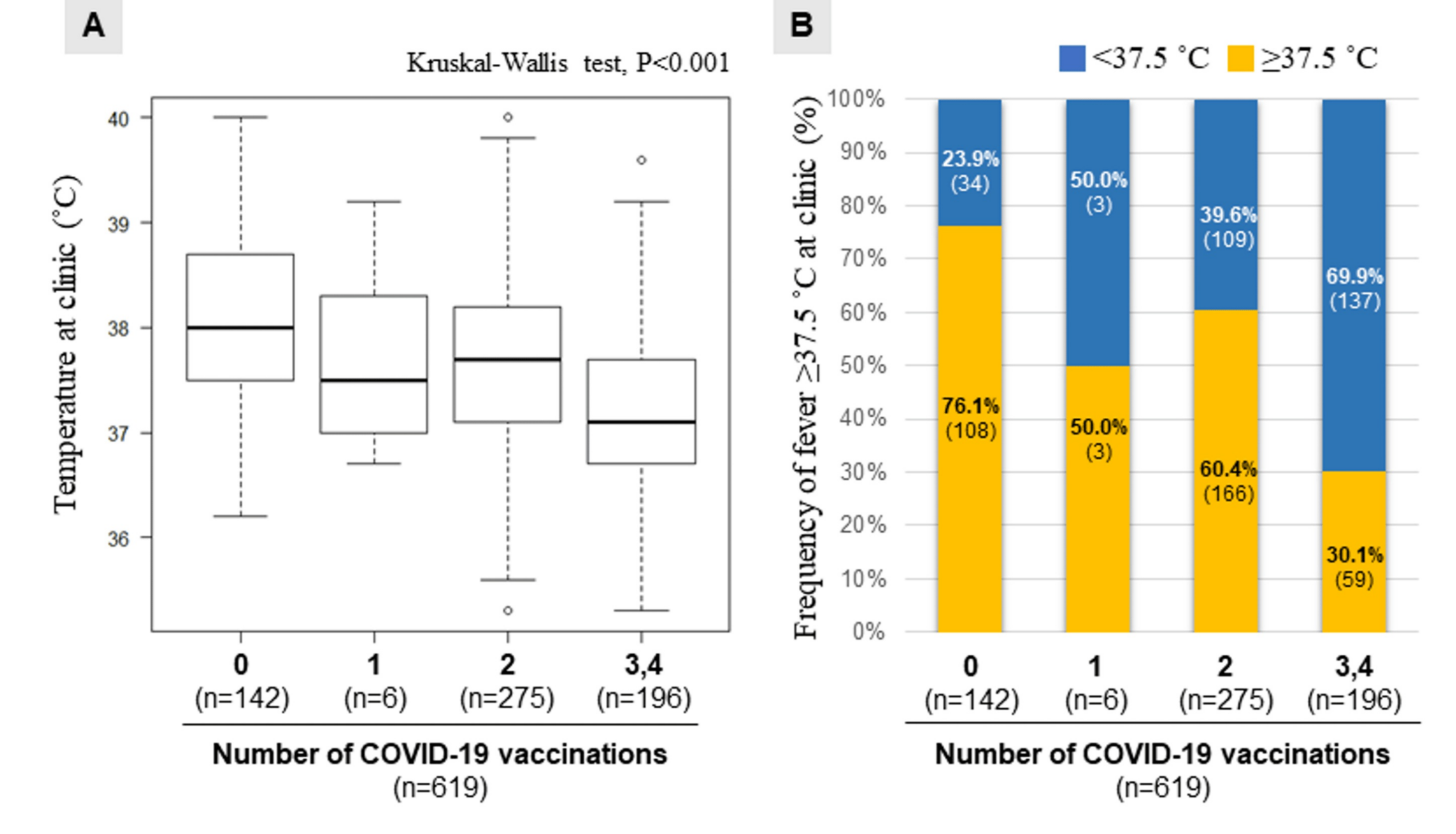
Impact of number of COVID-19 vaccinations on fever (A) The temperature at the clinic decreased as the number of vaccinations increased; (B) The frequency of fever (37.5°C or higher) markedly decreased as the number of vaccinations increased There is a data deficit of eight fevers COVID-19, coronavirus disease 2019

**Figure 4:**
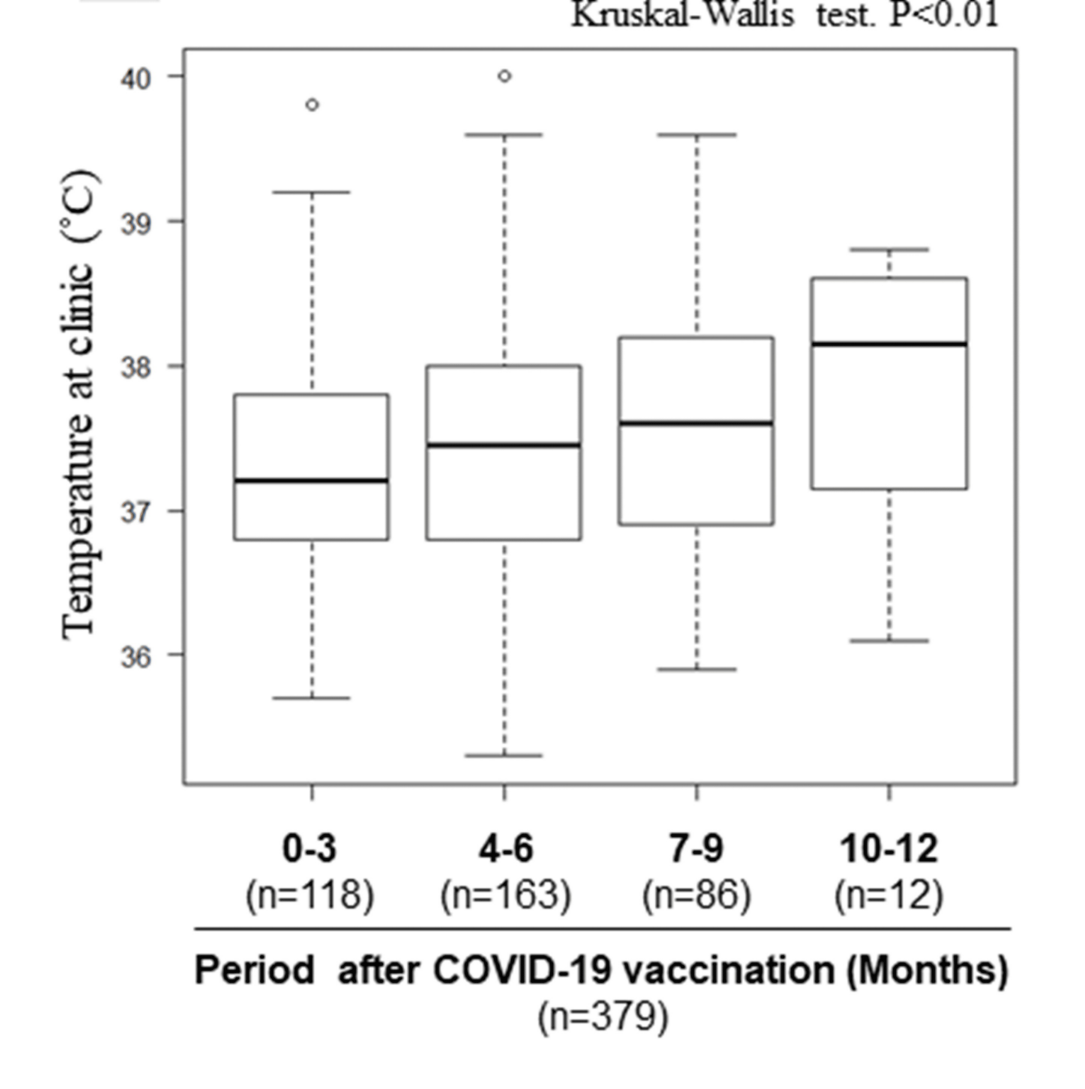
Impact of duration of time since COVID-19 vaccination on fever. As duration from last COVID-19 vaccination increased, the temperature at the clinic increased. The data excludes patients who did not remember the duration of time since their latest vaccine/number of vaccines. There is a data deficit of eight fevers. COVID-19, coronavirus disease 2019

The COVID-19 vaccinated group had a lower temperature at the clinic compared to the non-vaccinated group (37.4°C (IQR 36.8, 38.1) vs. 38.0°C (37.5, 38.7), P<0.001). The impact of COVID-19 vaccination on fever is shown in Figure 2. The temperature at the clinic and the frequency of fever decreased significantly as the number of vaccinations increased (Figure 3). Notably, fever was the most common manifestation in the non-vaccinated group (76%, n=108/142), whereas only 30% (59/196) of those who received three or more COVID-19 vaccinations experienced fever. Moreover, it was seen that temperature was related to the time duration after COVID-19 vaccination and increased as the duration became more (Figure 4).

**Figure 5:**
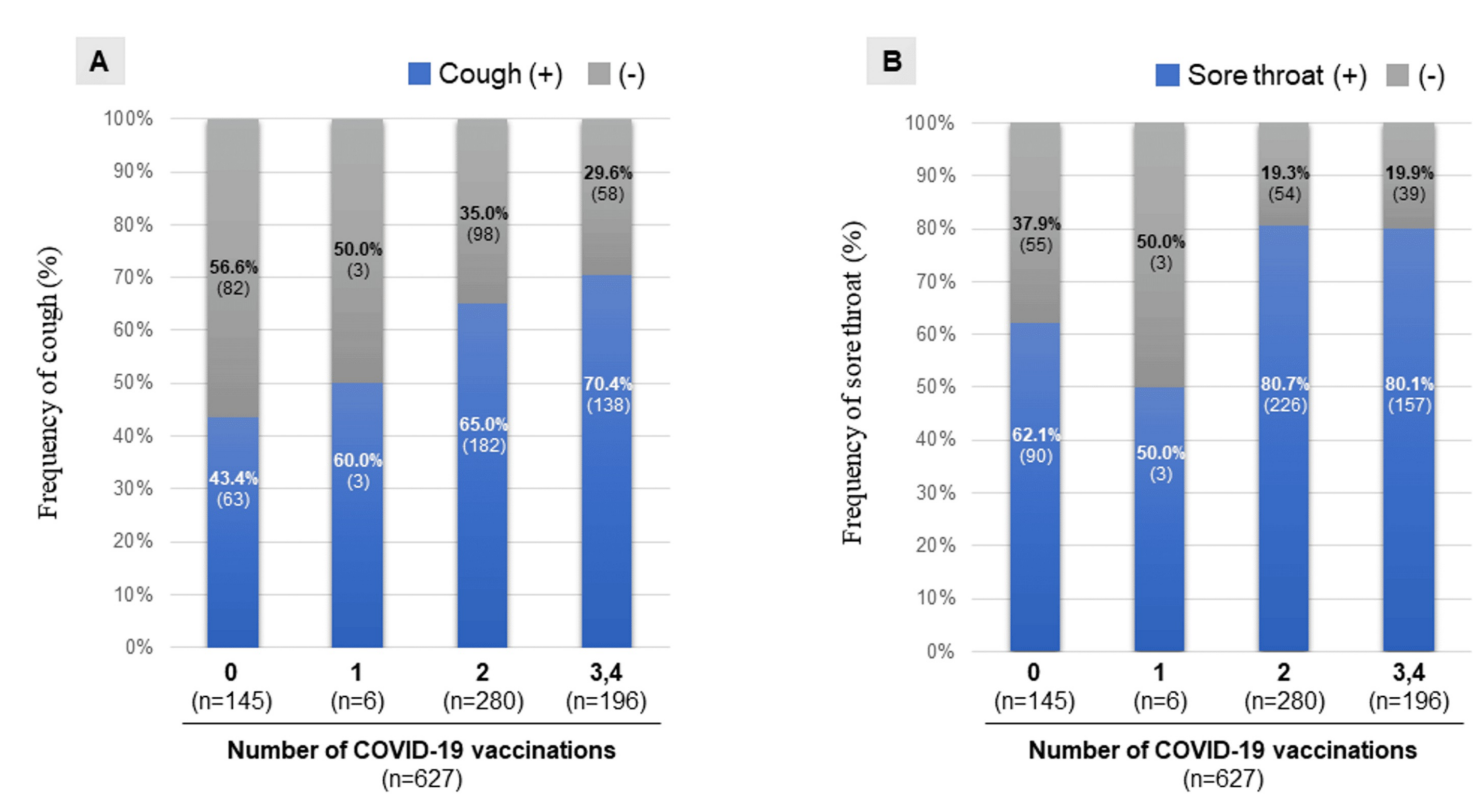
Relationship between cough (A) or sore throat (B) and number of COVID-19 vaccinations. COVID-19, coronavirus disease 2019

In the COVID-19 vaccinated group, the frequency of most manifestations, including fever, was reduced compared to the non-vaccinated group. Conversely, sore throat and cough were more common in the COVID-19 vaccinated group compared to the non-vaccinated group (Table 1). Furthermore, the frequency of both cough and sore throat increased with increasing COVID-19 vaccination frequency (Figure 5).

Multivariate analyses, adjusted for age, gender, the number of days since symptom onset, and the Omicron wave, showed that a history of COVID-19 vaccination was an independent factor associated with a lower frequency of fever and a higher frequency of cough or sore throat (Table 2).

**Table 2:** Impact of COVID-19 vaccination on fever, cough, sore throat manifestations CI, confidence interval; COVID-19, coronavirus disease 2019

Characteristics	Univariate analysis		Multivariate analysis	
	Odds ratio (95% CI)	p-value	Odds ratio (95% CI)	p-value
Fever (≥37.5 ˚C at clinic)				
Age, years	0.97 (0.96-0.98)	<0.001	0.98 (0.97-0.99)	<0.001
Sex, male	1.44 (1.06-1.96)	<0.05	1.36 (0.96-1.93)	0.081
Time from symptom onset to hospital visit, days	0.60 (0.52-0.69)	<0.001	0.60 (0.52-0.70)	<0.001
Omicron wave	0.54 (0.28-1.04)	0.067	0.36 (0.12-1.09)	0.07
History of COVID-19 vaccination	0.29 (0.19-0.44)	<0.001	0.38 (0.24-0.61)	<0.001
Cough				
Age, years	1.02 (1.01-1.03)	<0.001	1.01 (1.00-1.03)	<0.01
Sex, male	0.89 (0.65-1.21)	0.44	1.02 (0.73-1.42)	0.93
Time from symptom onset to hospital visit, days	1.22 (1.08-1.38)	<0.01	1.18 (1.04-1.35)	<0.05
Omicron wave	1.74 (0.94-3.24)	0.078	1.02 (0.42-2.48)	0.96
History of COVID-19 vaccination	2.67 (1.83-3.90)	<0.001	2.37 (1.57-3.59)	<0.001
Sore throat				
Age, years	1.00 (0.99-1.01)	0.38	0.99 (0.98-1.00)	0.095
Sex, male	0.65 (0.45-0.92)	<0.05	0.67 (0.46-0.98)	<0.05
Time from symptom onset to hospital visit, days	1.14 (1.00-1.31)	0.058	1.20 (1.03-1.40)	<0.05
Omicron wave	4.26 (2.27-8.00)	<0.001	2.58 (1.06-6.28)	<0.05
History of COVID-19 vaccination	2.42 (1.62-3.61)	<0.001	2.10 (1.35-3.28)	<0.01

## Discussion

Using data from the outpatient fever clinic in our hospital, we found the following outcomes: (i) Those who received a COVID-19 vaccination had a lower frequency of fever compared to those who were not vaccinated, (ii) Fever, a major symptom suspicious for SARS-CoV-2 infection, may be masked in vaccinated individuals, but cough and sore throat are more frequently present, and (iii) The frequency of cough and sore throat was higher in vaccinated patients than in non-vaccinated patients.

Fever as a common symptom of SARS-CoV-2 infection

COVID-19 symptoms often appear 2-14 days after infection with SARS-CoV-2. Symptoms of SARS-CoV-2 infection vary from person to person and can include fever or chills, cough, shortness of breath, sore throat, headache, and runny nose. Fever is one of the common symptoms of COVID-19; reported frequencies of fever in COVID-19-infected individuals range from 49.1% to 59% [6-10]. Therefore, checking for fever has a high clinical impact in diagnosing COVID-19. In this study, fever was present in most non-vaccinated patients, while it was markedly suppressed in vaccinated patients. Furthermore, our study demonstrated that the frequency of fever varies with the number of vaccinations and the time duration since vaccination, and these factors should be considered. Thompson et al. previously reported that COVID-19 vaccinees complained significantly less frequently of fever and chills compared to non-vaccinated individuals [11]. Their study also suggested that the attenuation of COVID-19 viral RNA load by vaccination might contribute to the symptom-relieving effect of the vaccine.

Given that vaccinated patients in this study also experienced suppression of many other symptoms, the diagnosis of SARS-CoV-2 infection might be overlooked. Cough and sore throat are major symptoms of SARS-CoV-2 infection, in addition to fever. These symptoms were found at a high frequency even in COVID-19-vaccinated individuals, as seen in this study. Therefore, the results of this study suggest that sore throat and cough could replace fever as key symptoms of SARS-CoV-2 infection in COVID-19-vaccinated individuals. Symptoms may vary depending on the number of days since onset. In the present study, however, multivariate analyses, even when the number of days since symptom onset was incorporated into the model, showed that a history of COVID-19 vaccination was an independent factor associated with a lower frequency of fever and a higher frequency of cough or sore throat.

Strategies to prevent the spread of SARS-CoV-2 infection in the general population

Since COVID-19 is known to be highly contagious, close contact with infected individuals should be avoided [10,12,13]. In particular, the elderly and those at higher risk for severe SARS-CoV-2 infections, such as individuals with hypertension and obesity, should keep the following three points in mind during epidemics as a precaution against infection: (i) Avoid poorly ventilated areas, (ii) Avoid crowded places, and (iii) Avoid close proximity conversations. Policies for SARS-CoV-2 infection control vary from country to country. In Japan, if a COVID-19 test is positive, social isolation and maintaining distance are crucial to preventing the spread of infection. Therefore, if SARS-CoV-2 infection is suspected, confirmation with a COVID-19 test kit is the first step in preventing further transmission. Acquiring knowledge of COVID-19 symptoms, regardless of vaccination status, plays an important role in prevention for the general population. Our results could contribute to enhancing this knowledge.

Unfavorable potential impact on symptoms following COVID-19 vaccination

Vaccine-associated enhanced disease (VAED) or antibody-dependent enhancement (ADE) following vaccination against severe acute respiratory syndrome (SARS) and Middle East respiratory syndrome (MERS) coronaviruses have been reported both in vivo and in vitro [14-16]. Individuals experiencing VAED or ADE may develop infections with more severe symptoms compared to non-vaccinated individuals, making these phenomena potential risks following COVID-19 vaccination. The underlying mechanisms are not fully understood, but the involvement of abnormal macrophages induced by the vaccines has been suggested [16]. Although VAED or ADE caused by COVID-19 vaccination has not been proven in clinical settings so far, recent studies have reported that IgG4 antibodies induced by repeated mRNA vaccination may suppress the immune system [17]. This phenomenon might contribute to the increased symptomatic appearance of sore throat and cough observed in this study. The present study demonstrated that the frequency of cough and sore throat increased with increasing number of vaccinations. It supports the hypothesis that repeated mRNA vaccination may cause immunosuppression. To verify these concerns, the long-term impact of COVID-19 vaccination needs to be carefully examined.

Limitations

There are several limitations to this study. The first major limitation is its retrospective, single-center study design. Therefore, the findings would need to be prospectively validated in other regions and hospitals. Second, symptoms may be influenced by new mutant strains of COVID-19. Different symptoms of SARS-CoV-2 infection have been reported before and after the emergence of the Omicron strain [18]. Although our study showed less fever and more cough and sore throat in vaccinated individuals, regardless of the Omicron wave, re-validation may be necessary as new variants become prevalent. Third, the COVID-19 vaccines used in this study include mRNA vaccines manufactured by Pfizer and Moderna/Takeda and a viral vector vaccine manufactured by Oxford/AstraZeneca. While almost all individuals in our community are vaccinated with mRNA vaccines, the exact vaccine administered to individual participants in this study is unknown. Vaccines with different mechanisms or produced by other companies might yield different results. Fourth, while this study observed that COVID-19 vaccination was associated with a higher frequency of symptoms such as cough and sore throat compared to non-vaccinated individuals, the severity of these symptoms was not assessed. Therefore, further investigation into the severity of symptoms is warranted. Moreover, the different frequency of cough and sore throat may be due to changes in the COVID-19 strain over time. Since the COVID-19 strain continues to change, it should be verified in the future. Lastly, the possibility that vaccination delays the onset of fever cannot be ruled out. Since we do not have data on the transition of fever after an outpatient visit, prospective studies following fever outpatient visits are needed to verify this issue.

## Conclusions

With the development of vaccines and antiviral agents against SARS-CoV-2 infection, COVID-19 has become somewhat controllable, with a decrease in disease severity and case-fatality rate. However, the high infectivity of COVID-19 can still cause an explosive spread of infection, adversely affecting social functions, particularly the healthcare system. Therefore, early diagnosis and social isolation to avoid close contact with others are crucial for preventing the spread of SARS-CoV-2 infection. This study demonstrated that vaccination alleviates symptoms after SARS-CoV-2 infection, but there is a concern about the potential for unawareness of SARS-CoV-2 infection due to symptom masking. This could lead to delayed detection of SARS-CoV-2 infection; thus, the possibility of infection must be considered in COVID-19 vaccinees even in the absence of fever. In contrast, sore throat and cough were observed at high rates even in COVID-19-vaccinated individuals; thus, testing is recommended when these symptoms are present.
